# Non-targeted Metabolite Profiling and Scavenging Activity Unveil the Nutraceutical Potential of Psyllium (*Plantago ovata* Forsk)

**DOI:** 10.3389/fpls.2016.00431

**Published:** 2016-04-05

**Authors:** Manish K. Patel, Avinash Mishra, Bhavanath Jha

**Affiliations:** Division of Marine Biotechnology and Ecology, Central Salt and Marine Chemicals Research InstituteBhavnagar, India

**Keywords:** antioxidant, bioactivity, isabgol, medicinal plant, metabolomics

## Abstract

Non-targeted metabolomics implies that psyllium (*Plantago ovata*) is a rich source of natural antioxidants, PUFAs (ω-3 and ω-6 fatty acids) and essential and sulfur-rich amino acids, as recommended by the FAO for human health. Psyllium contains phenolics and flavonoids that possess reducing capacity and reactive oxygen species (ROS) scavenging activities. In leaves, seeds, and husks, about 76, 78, 58% polyunsaturated, 21, 15, 20% saturated, and 3, 7, 22% monounsaturated fatty acids were found, respectively. A range of FAs (C12 to C24) was detected in psyllium and among different plant parts, a high content of the nutritive indicators ω-3 alpha-linolenic acid (57%) and ω-6 linoleic acid (18%) was detected in leaves. Similarly, total content of phenolics and the essential amino acid valine were also detected utmost in leaves followed by sulfur-rich amino acids and flavonoids. In total, 36 different metabolites were identified in psyllium, out of which 26 (13 each) metabolites were detected in leaves and seeds, whereas the remaining 10 were found in the husk. Most of the metabolites are natural antioxidants, phenolics, flavonoids, or alkaloids and can be used as nutrient supplements. Moreover, these metabolites have been reported to have several pharmaceutical applications, including anti-cancer activity. Natural plant ROS scavengers, saponins, were also detected. Based on metabolomic data, the probable presence of a flavonoid biosynthesis pathway was inferred, which provides useful insight for metabolic engineering in the future. Non-targeted metabolomics, antioxidants and scavenging activities reveal the nutraceutical potential of the plant and also suggest that psyllium leaves can be used as a green salad as a dietary supplement to daily food.

## Introduction

Psyllium (*Plantago ovata* Forsk) is an annual, herbaceous, medicinal plant belonging to the Plantaginaceae family and is widely cultivated in tropical regions of the world, such as India, Iran, Egypt, China, Korea, Japan, etc. (Chevallier, 1996). India is the largest producer of psyllium, which has been used since ancient times as a traditional herbal medicine in ayurvedic and allopathic preparations (Kirtikar and Basu, 1918). Its husk (commonly known as isabgol) and seed contain mucilaginous compounds of medicinal value and psylliums are used as a thickening agent in the pharmaceutical industry for manufacturing tablets. The husk is the white membranous structure covering the seed, which is a laxative and is particularly beneficial as a prophylactic in the treatment of bowel obstructions, constipation, diarrhea and dysentery (Chevallier, 1996).

*Plantago ovata* plant parts (leaves, seeds, and husks) are rich in bioactive compounds and different primary and secondary metabolites (Talukder et al., 2015). Among these, the most abundant compounds are fatty acids, amino acids, polyphenols, and flavonoids. One member of the Plantaginaceae contains an unusual hydroxy fatty acid, 9-hydroxy-*cis*-11-octadecenoic acid, which is an isomer of ricinoleic acid (Ahmad et al., 1980). Metabolites such as polyphenols and flavonoids are produced by secondary metabolism processes of *Plantago*, and possess a prominent antioxidant activity (Samuelsen, 2000; Beara et al., 2009). Polyphenols are considered to be very effective in the treatment of different types of neurodegenerative diseases, cardiovascular diseases and cancer, and are also involved in antioxidant activities, such as the scavenging of 2,2-azinobis-(3-ethylbenzothiazoline-6-sulphonic acid; ABTS), 2,2-diphenyl-1-picrylhydrazyl (DPPH) and hydroxyl radicals (Balasundram et al., 2006; Shahidi and Ambigaipalan, 2015). Many biological activities have been found associated with flavonoids and phenolic acids, such as anti-microbial, anti-hepatotoxic, anti-osteoporotic, anti-ulcer, immunomodulatory, anti-proliferative, and apoptotic activities (Al-Fayez et al., 2006; Gould and Lister, 2006; Grotewold, 2006). Most plant species have a robust defense system against reactive oxygen species (ROS)-induced oxidative stress (Niki et al., 1994; Chaturvedi et al., 2014; Tiwari et al., 2015). ROS can play a key role in cell damage by lipid peroxidation of membrane lipids (Braca et al., 2002; Joshi et al., 2013; Patel et al., 2015). Lipid peroxidation is an oxidative alteration of fatty acids (in the cellular membrane) that produces several types of scavenging free radicals (Niki et al., 1994). When ROS attack proteins, they generate protein carbonyls and other modifications in amino-acid residues, resulting in the destruction of protein function (Khan et al., 2010). Studies on medicinal plants, herbal plants and vegetables have indicated the presence of free-radical scavenging and antioxidant compounds, such as flavonoids, phenolics, terpenoids, saponines, coumarins, cardiac glycosides, tannins, and proanthocyanidins (Saeed et al., 2012). Previously, many plant extracts have been examined for different ROS-scavenging activities, including DPPH, superoxide, nitric oxide, reducing power and for phenolic and flavonoid content and total antioxidant activity (Mishra et al., 2015; Pandey et al., 2015). The use of medicinal plants with high constituents of antioxidant and free-radical scavenging compounds has been proposed as an effective therapeutic approach against hepatic and oxidative damage (Gould and Lister, 2006; Embuscado, 2015).

Plant metabolomics (metabolic profiling) has become an invaluable tool to study all the metabolites of plant tissues that have distinct chemical properties (Jorge et al., 2015). Plants possess the highest metabolic network complexity of all living organisms (Dersch et al., 2016). Metabolites are not only end products; they are also intermediates and substrates of metabolic processes and contribute toward plant adaptation. It is estimated that the plant kingdom alone is responsible for the synthesis of more than 200,000 metabolites, which are involved in various cellular processes (Pichersky and Gang, 2000; Fiehn, 2002). In the past decade, many analytical tools, such as gas chromatography mass spectrometry (GC–MS), high-performance liquid chromatography (HPLC), and liquid chromatography mass spectrometry (LC–MS) have been widely used within metabolomics for the identification and quantification of metabolites from different plant tissues. Metabolomics is the newest high-throughput technology for the qualitative and quantitative analysis of metabolites (Khakimov et al., 2014). Metabolite analysis helps to elucidate the function and pathways involved in the production of pharmacologically active compounds from plants (Rischer et al., 2006). Comprehensive metabolite analysis provides a useful insight into the existing metabolic pathways and also uncovers the network of metabolic pathways that are involved in different responses to specific stresses to be identified (Töpfer et al., 2015). Cross talk occurs between metabolites and environmental stress, which maintains the physio-biochemical status of the plant (Pandey et al., 2015). Furthermore, metabolite analysis is also emerging as a key tool in drug discovery processes (Fillet and Frédérich, 2015).

Psyllium is globally popular as a laxative, it is considered as a potential source of dietary supplementation and possesses important biological antioxidant and anti-inflammatory properties (Samuelsen, 2000; Beara et al., 2009). Studying the effect of organic additives in plant polyphenol accumulation is extremely important. No information is available to date concerning the metabolomics of this important plant. Therefore, this study was carried out to perform metabolic profiling and to characterize the antioxidant scavenging activities from different plant parts, e.g., leaves, seeds, and husks. Additionally, the total phenolic and flavonoid content was also estimated, a phytochemical analysis was performed, and a potential flavonoid biosynthesis pathway was inferred. This study provides useful insight into the metabolic responses of different plant parts of *P. ovata*, which reveal the potential for the plant to be used as a dietary supplement and in the nutraceutical industry.

## Materials and Methods

### Plant Material

**FIGURE 1:**
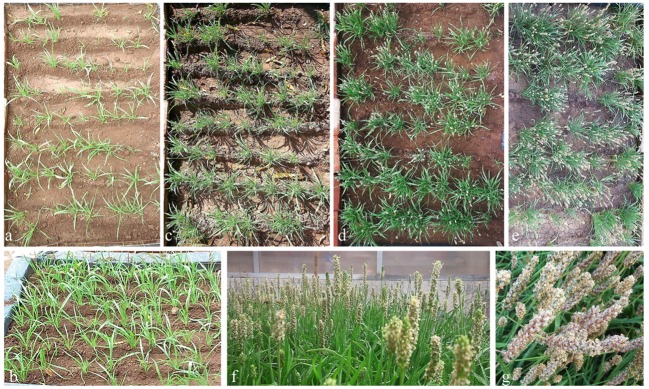
**Psyllium plants grown in plots under natural agro-climatic condition.** Plants were grown under natural agro-climatic conditions in a field. Plant growth status at 15 days **(a)**, 40 days **(b)**, 60 days **(c)**, 90 days **(d)**, 100 days **(e)**, and 120 days **(f)** of growth. Plants showing seed maturity at 120 days **(g)** of growth.

Seeds of *P. ovata* were procured from Seed Spices Research Station, Jagudan, Mehsana, Gujarat, India and were germinated in a plot (**Figure 1**) containing garden soil, under natural agro-climatic field conditions from November, 2014 to March, 2015 (Jat et al., 2015). A plot consisted of eight rows and each row contained about eight plants. The plants were irrigated every alternate day with tap water. Leaves from 3-months-old plants, mature seeds and husk were harvested and immediately used for further study.

### Lipid Extraction and Fatty Acid Profiling

Total lipid was extracted from 1 g biomass of leaves, seeds, and husk using the solvent chloroform–methanol (v/v, 1:2) extraction method followed by washing with an equal volume of phosphate buffer (pH 7.5), and stored at -20°C for further analysis (Bligh and Dyer, 1959). The corresponding fatty acid methyl esters (FAMEs) were prepared by transmethylation (Kumari et al., 2013). Lipid samples were transmethylated by adding NaOH (v/v, 1% in methanol; 1 ml) in the vessels, followed by heating at 55°C for 15 min. Thereafter, 2 ml methanolic HCl (v/v, 5%) was added and the vessels were further heated at 55°C for 15 min. Derivative FAMEs were extracted in aqueous hexane (v/v, 1:2; 3 ml), dried under N_2_ and dissolved in hexane (200 μl).

Samples of FAME of each plant part were analyzed by a GC coupled with a mass spectrometer (GCMS-QP2010, Shimadzu, Japan) equipped with an auto-sampler (AOC-5000) using a RTX 5MS capillary column (60 m length, 0.25 mm diameter, and 0.50 μm thickness; Rastek, USA). Helium (99.9% purity) gas was used as a carrier gas with a flow rate of 1 ml min^-1^ and a pre-column pressure of 49.7 kPa. The initial column temperature was 40°C for 3.0 min, followed by 5°C min^-1^ increments up to 230°C and finally, 230°C held for 40 min. The injection volume, temperature, and total analysis time were 1 μl, 240°C and 67 min, respectively. The mass spectrometer operated in ionization mode, with electron impact at 70 eV and the temperature of the ion sources and quadrupole was 200°C (Mishra et al., 2015). The limit of detection (LOD) and limit of quantitation (LOQ) of the instrument were established (Supplementary Table S1) and FAME peaks were acquired over the 40–400 m/z range. The MS peaks of samples were compared with the retention times of standards (FAME Mix C4-C24, Supelco, USA and 7-hexedecenoic acid methyl ester, Cayman Chemicals, USA) by GCMS analysis and were quantified by area normalization.

The total content of saturated fatty acids (SFAs) and unsaturated fatty acids [monounsaturated fatty acids (MUFA) and polyunsaturated fatty acids (PUFA)] were determined by summation of the percentage quantity of the corresponding fatty acids. Unsaturation index (UI; Poerschmann et al., 2004) and degree of unsaturation (DU; Yu et al., 2012) were calculated using the following equation

D⁢e⁢g⁢r⁢e⁢e⁢ o⁢f⁢ u⁢n⁢s⁢a⁢t⁢u⁢r⁢a⁢t⁢i⁢o⁢n⁢ (D⁢U)=(M⁢U⁢F⁢A,w%)+2⁢(P⁢U⁢F⁢A,w%)

                                 Unsaturation index (UI) = Σ⁢(U⁢F⁢A,w% ×n⁢u⁢m⁢b⁢e⁢r⁢  o⁢f⁢ d⁢o⁢u⁢b⁢l⁢e⁢ b⁢o⁢n⁢d⁢s)⁢. 

### Amino Acid Profiling

Plant samples (5 mg each dried and powdered; leaves, seed and husk) were hydrolysed in a glass vessel with HCl (6 N, 500 μl). Glass vessels were made air-free by flushing with N_2_ and were sealed. The samples were hydrolysed at 110°C for 24 h in a hot-air oven. After hydrolysis, the vessels were broken and the samples were vacuum-dried in a desiccator. The mixture of ethanol–water–TEA (v/v, 2:2:1; 500 μl) was added to the vessels, which contained hydrolysed samples and an amino acid standard (10 μl, AAS18, Sigma, USA) for neutralization. The samples were mixed properly by vortexing and were then vacuum-dried. Thereafter, samples were derivatised by adding a mixture of ethanol–water–TEA–PITC (v/v, 7:1:1:1; 500 μl) and were vortexed for proper mixing. The reaction mixture was kept at room temperature for 20 min, was vacuum-dried and finally dissolved in 400 μl Na_2_HPO_4_ buffer (5 mM, pH 7.4) containing acetonitrile (v/v, 5%). Samples were filtered with a 0.2 μm membrane and the amino acid composition was analyzed using HPLC (Kwanyuen and Burton, 2010).

The standard (AAS18, Sigma, USA) and samples were injected and analyzed with a HPLC system (Waters Alliance model, 2996-seperation module with an auto-sampler, USA) equipped with a Luna-C18 reversed-phase (5.0 μm, 4.6 mm × 150 mm, Phenomenex, USA) column (Mishra and Jha, 2009; Kwanyuen and Burton, 2010). The amino acids were separated and eluted by a gradient resulting from mixing eluents A and B. Eluent A consisted of 150 mM CH_3_COONa.3H_2_O, 0.05% (v/v) TEA and 6% (v/v) acetonitrile, pH 6.4, whereas eluent B consisted of acetonitrile:water (v/v, 6:4). Both eluents were properly mixed and filtered through a 0.2 μm membrane. The flow rate was 1 ml min^-1^ throughout and the gradient consisted of the following profiles: 100% A at the start, 80% A and 20% B at 5.5 min, 54% A and 46% B at 10 min, 100% B at 10.5–12.5 min, 100% A at 13 min. The PITC-derivatised amino acids were eluted from the column and recorded at 254 nm. The relative proportion of the peak area was calculated to estimate the amino acid composition per gram dry weight of plant samples.

### Screening of Phytochemicals

A phytochemical analysis of psyllium plant parts (leaves, seeds, and husk) was performed to determine the presence of alkaloids, terpenoids, saponins, coumarins, flavonoids, cardiac glycosides, and tannins. To test for the presence of alkaloids in the plant parts, 0.1 g sample was mixed with 4 ml 1% HCl in a glass tube, heated in a water bath, and filtered. A 2 ml aliquot of filtrate was treated with Dragendroff’s reagent (potassium bismuth) and the appearance of turbidity or precipitation demonstrated the presence of alkaloids (Harborne, 1973).

The presence of terpenoids in the plant parts was confirmed by the appearance of a reddish brown interface following mixing 1 mg ml^-1^ aqueous extract with 2 ml chloroform, followed by the addition of 3 ml concentrated H_2_SO_4_ (Harborne, 1973). Saponins were tested by mixing about 20 mg sample with distilled water (15 ml), incubating in a boiling water bath for 5 min and filtering. The filtrate was diluted with 5 ml water and was vortexed vigorously to form froth. The ability of saponins to produce an emulsion with oil was tested by mixing three drops of olive oil with the froth (Harborne, 1973).

To test for the presence of coumarins, about 30 mg sample was dropped onto filter paper moistened with 1 N NaOH. The filter paper was placed in a test tube and was incubated in a boiling water bath for a few minutes. The filter paper was examined under UV light and a yellow florescence indicated the presence of coumarins (Trease and Evans, 1989). The presence of flavonoids was demonstrated by the appearance of a yellow color (Sofowora, 1993), when 3 mg filtered aqueous sample (5 ml water) was mixed with 2 ml dilute ammonia solution followed by a few drops of concentrated H_2_SO_4_.

To test for the presence of cardiac glycosides, 10 mg plant sample dissolved in methanol was mixed with 2 ml of glacial acetic acid and one drop of FeCl_3_ solution was added. If cardiac glycosides were present, a brown ring appeared at interface after adding 1 ml of concentrated H_2_SO_4_ to the above mixture (Trease and Evans, 1989). To test for tannins 10 mg filtered aqueous plant sample (in 5 ml boiled water) was mixed with few drops of 0.1% FeCl_3_ and the appearance of a brownish green or blue-black color confirmed the existence of tannins (Sofowora, 1993).

### Extract Preparation

Plant sample (leaves, seeds, and husk; 10 g) were ground in liquid N_2_, transferred to aqueous methanol (v/v, 70%) and kept overnight for extraction. The mixture was centrifuged at 8000 rpm for 10 min and the supernatant was collected. The extraction (in aqueous methanol) was repeated twice. Collected supernatants were concentrated in a rotary evaporator (150–100 mbar at 37°C) and were lyophilised. The dried residue was stored at -20°C until use.

To determine different activities (antioxidant and scavenging) and contents (phenolic and flavonoid), dried residue was solubilised in distilled water, and absorbance readings of samples (plant extracts) were compared with a standard curve, which was created by the same method, using known amounts of the corresponding standard. All tests were performed in triplicate and values were expressed as mean ± SE.

### Total Phenolic Content

Total phenolic content of the plant extracts was determined by the Folin-Ciocalteu (FC) reagents using gallic acid as a standard (Hazra et al., 2008; Mishra et al., 2015). Different concentrations of the plant extracts (50–1000 μg ml^-1^) were mixed with 2.5 ml 0.2 N Folin–Ciocalteu reagent (Sigma-Aldrich, USA) and were incubated for 5 min, followed by the addition of 2 ml sodium carbonate (Na_2_CO_3;_ 75 g l^-1^). The reaction mixtures were incubated for a further 90 min at room temperature. The absorbance was measured at 760 nm and the total phenolic content was calculated as mg ml^-1^ gallic acid per 100 mg extract from a standard curve.

### Total Flavonoid Content

To determine the total flavonoid content, different concentrations of plant extracts (50–1000 μg ml^-1^) were added to 0.3 ml NaNO_2_ (v/v, 5%) and were incubated for 5 min at room temperature. Thereafter, 0.3 ml AlCl_3_ (v/v, 10%) and 2 ml NaOH (1 M) were added. The reaction mixture was diluted with water and absorbance was measured at 510 nm and the total flavonoid content was calculated as mg ml^-1^ quercetin per 100 mg extract from a standard curve (Zhishen et al., 1999; Mishra et al., 2015).

### ABTS Scavenging Activity

Total antioxidant activity was measured by comparing the ABTS^+^ radical cation scavenging ability of plant extracts with that of the standard, trolox (Re et al., 1999; Hazra et al., 2008). The ABTS diammonium salt (7 mM) solution was mixed with potassium persulfate (2.45 mM) and the mixtures were incubated in the dark for 12–16 h at room temperature to generate ABTS^+^ radical cations. The ABTS radical cation solution was diluted with water for an initial absorbance of the solution of about 0.70 ± 0.02 at 735 nm. The radical cation scavenging activity was assessed using 1 ml of the diluted radical cation solution mixed with different concentrations of the plant extracts (50–300 μg ml^-1^) or the standard (1–5 μg ml^-1^ trolox). After incubation, the absorbance was measured at 734 nm. The percentage inhibition of absorbance was calculated and the activity of different extracts (leaves, seeds, and husk) was compared.

### DPPH Assay

Scavenging of the DPPH free radical was determined using trolox as a standard (Saeed et al., 2012). The DPPH stock solution (w/v, 0.024% in methanol) was diluted to make a working solution by adding methanol until an absorbance of 0.98 ± 0.02 at 517 nm was obtained. Different concentrations of plant extracts (50–300 μg ml^-1^) were mixed with 3 ml working stock solution and were incubated overnight at room temperature in the dark. The absorbance was measured at 517 nm and the radical scavenging activities of the plant extracts were estimated using the following equation:

$$Scavenging\,\;activity\,\;(\%)=[\frac{OD_{517}\,\;of\,\;control-OD_{517}\,\;of\,\;Extract}{OD_{517}\,\;of\,\;control}]\times 100.$$

### Reducing Power Assay

Different concentrations of the plant extracts (5–80 μg ml^-1^) or ascorbic acid (as a positive standard) were mixed with 1 ml phosphate buffer (0.2 M, pH 6.6), then 1 ml of K_3_Fe(CN)_6_ (10 mg ml^-1^) was added and the mixture was incubated at 50°C for 20 min in a water bath. After incubation, 1 ml trichloroacetic acid (100 mg l^-1^) was added to terminate the reaction. The reaction mixture was centrifuged at 8000 rpm for 10 min at room temperature and the supernatant was collected in a 5-ml tube. A 1-ml aliquot of the diluted supernatant was mixed with 0.2 ml freshly prepared FeCl_3_ (w/v, 0.1%) and was incubated for 10 min at room temperature. The absorbance was measured at 700 nm, the scavenging activity was measured and the reducing power was compared.

$$Scavenging\,\;activity\,\;(\%)=[\frac{OD_{700}\,\;of\,\;control-OD_{700}\,\;of\,\;Extract}{OD_{700}\,\;of\,\;control}]\times 100.$$

### Superoxide Assay

The activity of superoxide anion was measured by the reduction of nitro blue tetrazolium (NBT) according to a previously described method (Hazra et al., 2008). Superoxide anion radicals are generated by a non-enzymatic phenazine methosulphate/nicotinamide adenine dinucleotide (PMS/NADH) system. These superoxide radicals reduce NBT to a purple-colored formazan. The reaction mixture contained NBT (50 μM), PMS (15 μM), NADH (73 μM) in phosphate buffer (20 mM, pH 7.4) and various concentrations of the plant extracts (50–300 μg ml^-1^). The mixture was incubated for 5 min at room temperature and absorbance was measured at 562 nm. Quercetin was used as standard. The inhibition of superoxide anion radical generation was estimated using the following equation:

$$Scavenging\,\;activity\,\;(\%)=[\frac{OD_{562}\,\;of\,\;control-OD_{562}\,\;of\,\;Extract}{OD_{562}\,\;of\,\;control}]\times 100.$$

### Metabolites Profiling

Plant samples (leaves, seed, and husk; 100 mg) were ground and the total plant metabolites were extracted using a modified method (Mishra et al., 2015) by adding ice-cold aqueous methanol (v/v, 70%) followed by vortexing. The sample was placed in an ultrasonic water bath (MRC, Israel) at a frequency of 40 kHz for 1 h. The supernatant was collected after centrifugation (20000 rpm at 25°C for 10 min) and was filtered (0.2 μm membrane).

Metabolites were analyzed by LC coupled with TOF MS/MS (Micromass, Waters, USA) and were identified by comparing LC-TOF MS/MS peaks using the on-line METLIN database (Zhu et al., 2013). The LC MS/MS parameters were a source and desolvation temperature of 110 and 200°C respectively; 2.5 kV was applied to the electrospray capillary, the cone voltage was 25 V and nitrogen was used as the collision gas. Samples were directly injected to the ESI-MS at a 50 μl min^-1^ flow rate using a syringe pump, and the extracted metabolites were examined in negative-ion ESI/MS-MS mode. The scanning range was 0–1000 m/z, with an acquisition rate of 0.25 s and an inter-scan delay of 0.1 s. For peak integration, the background of each spectrum was subtracted, the data were smoothed and cantered and the peaks were integrated using the Mass Lynx software version 4.1 (Micromass, Waters).

### Pathway Analysis

A potential flavonoid biosynthesis pathway was predicted by *in silico* comparative and interactive pathway topology analysis using the metabolomic data (Xia et al., 2015). A total of 24 metabolites was used for the analysis and compounds with no match were excluded from the subsequent pathway analysis (Supplementary Table S2). The metabolome data were uploaded and subsequently compared with the KEGG pathway library of *Oryza sativa* japonica and *Arabidopsis thaliana* by over-representation analysis using Fishers’ exact test. The pathway topology was analyzed by a well-established node of centrality measures to estimate the node and a graph-based method was used to analyze the biological networks (Aittokallio and Schwikowski, 2006). The degree of centrality used for the comparison among different pathways was calculated. The node importance value was calculated from centrality measures and was further normalized by the sum of the importance of the pathway. The pathway was predicted among different pathways using the statistical *p*-values from enrichment analysis, which was further adjusted for multiple testing. The pathway was selected to show maximum hits, which is actually a matched number from the uploaded data. The significance of the analysis was calculated as a *p*-value from the enrichment analysis, as the *Holm p*-value, in which the *p*-value was adjusted by the Holm-Bonferroni method, as the *FDR p*-value by adjusting the *p*-value using the false discovery rate and finally, the impact value was calculated using pathway topology analysis (Xia et al., 2015).

### Statistical Analysis

All experiments were carried out three times and for each experiment, three biological replicates were performed (i.e., three plant samples per experiment). All data were subjected to analysis of variance (ANOVA); *p* < 0.05 was considered as the threshold for statistical significance and values were expressed as the mean ± SE (standard error of the mean). All lipid and fatty acid datasets were analyzed individually and in combination by principal component analysis (PCA) and respective heat maps were generated.

## Results

### Fatty Acid Composition

The total fatty acid content was 26.17 ± 1.35, 156.89 ± 4.39, and 28.50 ± 2.21 mg per gram FW of leaves, seeds, and husk, respectively (Supplementary Table S3). The total fatty acid (FA) content of leaves, seeds, and husk consisted of about 76, 78, 58% polyunsaturated, 21, 15, 20% saturated, and 3, 7, 22% MUFA, respectively (**Table 1**). The maximum degree of unsaturation (DU) was observed in seeds followed by in leaves and the husk, whereas the maximum UI was observed in leaves followed by in seeds and the husk. Among PUFAs, C18 PUFA dominated in all plant parts, and notably, C22 PUFAs and C20 PUFAs were not detected in leaves and seeds, respectively.

**Table 1:** Fatty acid profile of different psyllium plant parts.

Fatty acids	Name of fatty acids	Leaves	Seeds	Husk
**Lipid composition**				
ΣSFA		21.012 ± 0.2	14.46 ± 0.65	19.88 ± 0.46
ΣMUFA		2.86 ± 0.69	7.34 ± 0.71	21.58 ± 0.1
ΣPUFA		76.12 ± 0.52	78.2 ± 0.31	58.55 ± 0.39
Σ18 PUFA		75.06 ± 0.21	75.82 ± 1.39	48.25 ± 1.48
Σ20 PUFA		1.06 ± 0.12	nd	4.67 ± 0.12
ΣC22 PUFA		nd	2.38 ± 1.53	5.62 ± 1.53
UI		212.17 ± 3.44	185.5 ± 4.45	164.31 ± 7.79
DU		155.09 ± .39	163.74 ± 0.73	138.67 ± 0.84
n6/n3		0.31 ± 0.57	4.49 ± 0.57	6.36 ± 1.12
n9/n3		0.03 ± 0.29	0.5 ± 0.2	2.98 ± 0.48
n9/n6		0.1 ± 0.01	0.11 ± 0.02	0.47 ± 0.01
**Fatty acids composition**				
C12:0	Lauric acid	nd	nd	1.00 ± 0.06
C14:0	Myristic acid	nd	0.26 ± 0.01	0.25 ± 0.01
C14:1	Myristoleic acid	0.89 ± 0.08	nd	nd
C15:0	Pentadecanoic acid	0.12 ± 0.01	0.13 ± 0.01	0.1 ± 0.01
C15:1 (n-5)	*Cis*-10-pentadecenoic acid	nd	0.05 ± 0.004	nd
C16:0	Palmitic acid	13.7 ± 0.25	6.56 ± 0.63	12.22 ± 0.4
C16:1 (n-7)	Palmitoleic acid	0.12 ± 0.01	0.17 ± 0.02	0.1 ± 0.02
C17:0	Heptadecanoic acid	0.16 ± 0.01	0.22 ± 0.01	0.12 ± 0.01
C17:1 (n-7)	*Cis*-10-heptadecanoic acid	nd	0.13 ± 0.01	0.07 ± 0.01
C18:0	Stearic acid	5.27 ± 0.08	5.8 ± 0.06	4.83 ± 0.06
C18:1 (n-9)	Oleic acid	1.84 ± 0.36	nd	18.92 ± 0.36
C18:2 (n-6)	Linoleic acid	18.14 ± 0.57	63.58 ± 0.88	45.1 ± 1.31
C18:3 (n-3)	Alpha-linolenic acid [ALA]	56.92 ± 0.62	11.89 ± 0.89	2.73 ± 0.28
C18:3 (n-6)	Gamma-linolenic acid	nd	0.36 ± 0.01	0.41 ± 0.01
C20:0	Arachidic acid	0.4 ± 0.02	0.61 ± 0.03	0.57 ± 0.04
C20:1 (n-9)	*Cis*-11-eicosenoic acid	nd	6.98 ± 0.67	2.04 ± 0.67
C20:2	*Cis*-11,14-eicosadienoic acid	nd	nd	4.67 ± 1.20
C20:3 (n-3)	*Cis*-11,14,17-eicosadienoic acid	1.05 ± 0.16	nd	nd
C21:0	Heneicosanoic acid	nd	0.39 ± 0.04	0.4 ± 0.04
C22:0	Behenic acid	0.49 ± 0.01	0.24 ± 0.03	0.39 ± 0.03
C22:1 (n-9)	Erucic acid	nd	nd	0.43
C23:0	Tricosanoic acid	nd	0.08 ± 0.01	nd
C24:0	Lignoceric acid	0.87 ± 0.01	0.16 ± 0.01	nd

*SFAs, Saturated fatty acids; MUFA, mono unsaturated fatty acids; PUFA, poly unsaturated fatty acids; UI, unsaturation index; DU, degree of unsaturation; nd, not detected*.

**FIGURE 2:**
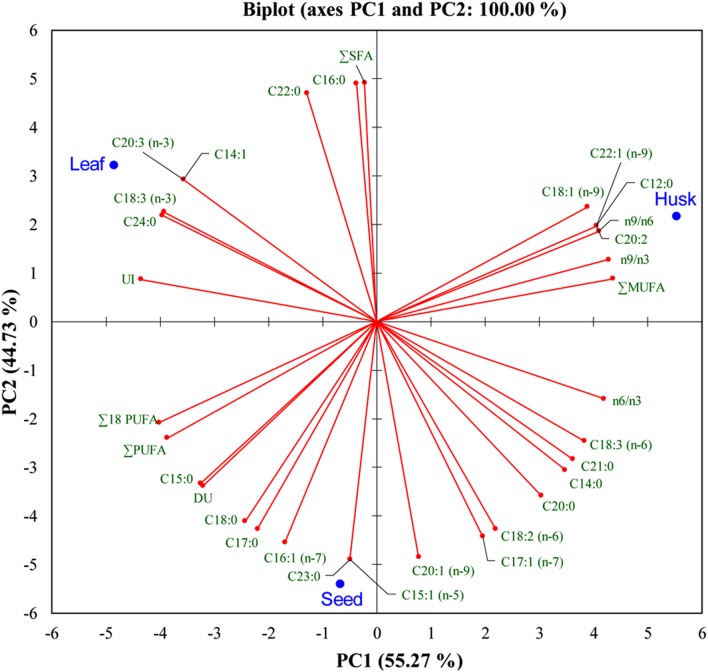
**Bi-plot of psyllium plant-part samples obtained from principal component analysis of data matrix of the total lipids and fatty acids according to **Table 1** with first two principal components**.

**FIGURE 3:**
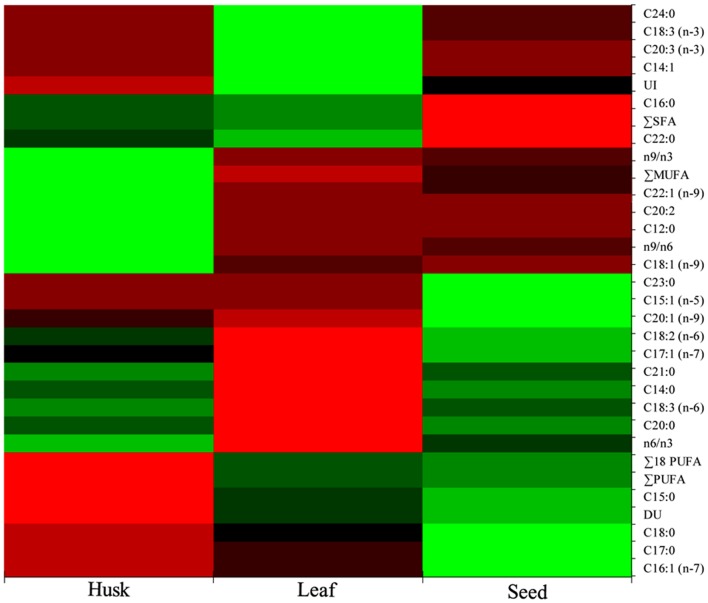
**Heat map of the total lipids and fatty acids composition showing spatial occurrence of lipids and fatty acids**.

A range of FAs (C14 to C24) was detected, with a predominance of alpha-linolenic acid (C18:3, n-3; 57%), linoleic acid (C18:2, n-6; 18%), palmitic acid (C16:0; 14%), and stearic acid (C18:0; 5%) in leaves. A dominance of linoleic acid (C18:2, n-6; 64%) was detected in seeds, together with alpha-linolenic acid (C18:3, n-3; 12%), *cis*-11-eicosenoic acid (C20:1, n-9; 7%), palmitic acid (C16:0; 6.5%) and stearic acid (C18:0; 6%) as major FAs. In the husk, there was a dominance of linoleic acid (C18:2, n-6; 45%) followed by oleic acid (C18:1, n-9; 19%), palmitic acid (C16:0; 12%), stearic acid (C18:0; 5%), and *cis*-11,14-eicosadienoic acid (C20:2; 5%). Myristoleic acid (C14:1; 0.9%) and *cis*-11,14,17-eicosadienoic acid (C20:3, n-3; 1%) were detected in only in leaves. Similarly, *cis*-10-pentadecenoic acid (C15:1, n-5; 0.5%) and tricosanoic acid (C23:0; 0.1%) were present only in seed, whereas three FAs; *cis*-11,14-eicosadienoic acid (C20:2; 5%), lauric acid (C12:0; 1%), and erucic acid (C22:1, n-9; 0.4%) were detected exclusively in the husk. Four FAs were not detected in leaves; myristic acid (C14:0), *cis*-10-heptadecanoic acid (C17:1, n-7), gamma-linolenic acid (C18:3, n-6), and heneicosanoic acid (C21:0); however, these were present in seeds and in the husk as minor FAs. One FA, oleic acid (C18:1, n-9), which was present in the husk as a major FA was not detected in seeds; similarly, lignoceric acid (C24:0,) was present in leaves and seeds, but was not detected in the husk. PCA indicated that the total lipid and fatty acid composition was significantly dependent on the plant parts (**Figure 2**) and the heat map showed the differential fatty acid composition (**Figure 3**).

### Amino Acid Constituents

**FIGURE 4:**
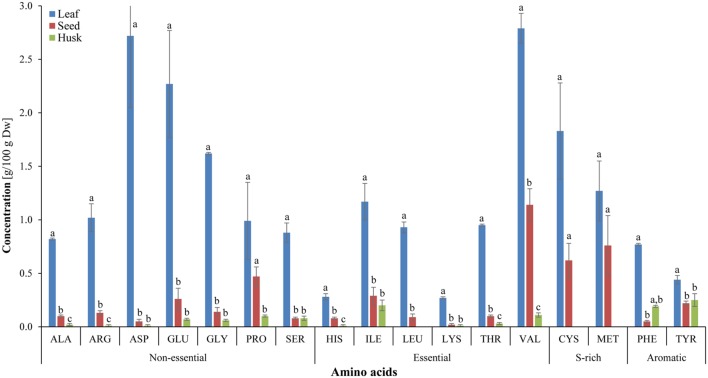
**Amino-acid composition of different psyllium plant parts**. Value represents the mean ± SE. Value represents the mean ± SE and values of each amino acids with different letters are significant different at *p* < 0.05.

In total, 17 amino acids were detected in leaves and seeds, whereas 14 amino acids were observed in the husk and were categorized as non-essential, essential, sulfur-rich and aromatic amino acids (**Figure 4**). Essential amino acids, isoleucine (1.17%), threonine (0.95%), leucine (0.93%), histidine (0.28%), and lysine (0.27%) were significantly higher in leaves compared to seeds and husks, in which contents of these essential amino acids were insignificant (0.1–0.01% of biomass). The highest levels of the essential amino acid valine was detected in leaves and seeds (about 3.0 and 1.0 g per 100 g biomass, respectively) followed by sulfur-rich amino acids. In contrast, aromatic amino acids were abundant in the husk compared to essential and non-essential amino acids. High amounts of almost all amino acids were detected in leaves and seeds compared to in the husk. The essential amino acids leucine and the sulfur-rich amino acids cysteine and methionine were not detected in the husk.

### Phytochemicals Assay, Total Phenolic and Flavonoid Contents

**FIGURE 5:**
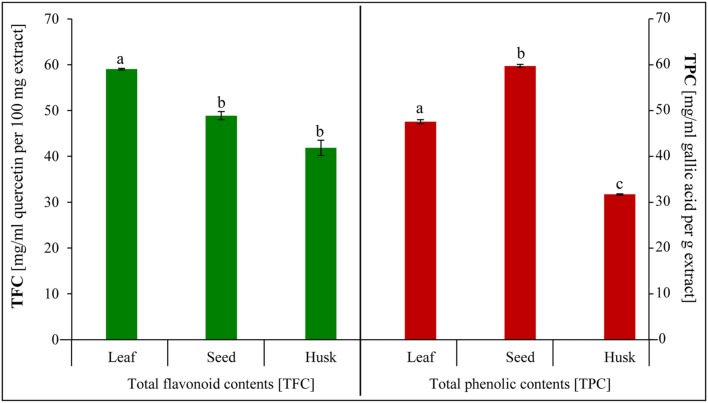
**Total phenolic and total flavonoid contents extracted from psyllium leaves, seeds, and husk.** Value represents the mean ± SE and values with different letters are significant different at *p* < 0.05.

Different edible plant parts (leaves, seeds, and husk) were screened to ascertain the occurrence of phytochemicals (**Table 2**). Alkaloids, flavonoids, cardiac glycosides, and tannins were detected in all plant parts. In contrast, terpenoids and saponines were absent in leaves, whereas coumarins were not detected in seeds and the husk. The total flavonoids was detected utmost in the leaves, followed by in seeds and the husk (**Figure 5**). Similarly, a high total phenolic content was observed in seeds followed by leaves and the husk.

**Table 2:** Phytochemicals assay of psyllium plant extract.

	Alkaloids	Terpenoids	Saponines	Coumarins	Flavonoids	Cardiac glycosides	Tannins
Husk	+	+	+	-	+	+	+
Leaf	+	-	-	+	+	+	+
Seed	+	+	+	-	+	+	+

### Scavenging and Reducing Activities

**FIGURE 6:**
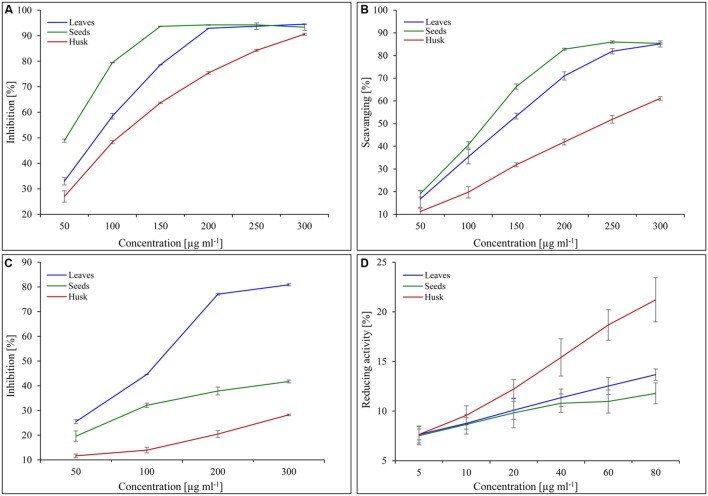
**Scavenging and reducing activities of psyllium extracts obtained from different plant parts.** Total antioxidant **(A)**, DPPH scavenging **(B)**, superoxide free radicals scavenging **(C)** and reducing activities **(D)** of psyllium leaves, seeds, and husk extracts. Value represents the mean ± SE.

The reducing capacity and scavenging activity of the plant extracts increased concomitantly with the extract concentration. The total antioxidant activity (expressed as the % inhibition of decolorisation of ABTS^.+^) was found to be the highest in the seed extract followed by in leaf and husk extracts (**Figure 6A**). About 150 μg seed extract showed 94% inhibition, whereas the same amount of inhibition was observed with 200 μg leaf extract. In contrast, lower total antioxidant activity was observed with husk extract and about 300 μg extract was required for 90% inhibition. Similarly, the maximum DPPH scavenging activity was found for seed extract, followed by leaf and husk extracts (**Figure 6B**). About 85% scavenging activity was observed with 250 and 300 μg seed and leaf extracts, respectively, whereas about 60% scavenging was found with 300 μg husk extract. The leaf extract (300 μg) showed the maximum (80%) superoxide scavenging activity, whereas about 40 and 30% scavenging activity was observed with seed and husk extracts (300 μg), respectively (**Figure 6C**). Notably, the maximum reducing activity (20%) was shown by the husk extract (80 μg), followed by that of leaves and seeds extracts (**Figure 6D**).

### Metabolites Profiling

Thirteen different metabolites were identified in leaf and seed extracts, whereas 10 metabolites were found in the husk (**Table 3**). In total, eight alkaloids and flavonoids were detected in the leaf extract followed by five in that of the seed and one in the husk extract. No alkaloids were present in the leaf extract, whereas an alkaloid pinidine (m/z 122.13) was detected in the husk extract and, two alkaloids, lunamarine (punarnavine; m/z 104.04) and hordatine B (m/z 545.3), were observed in the seed extract. A total of seven nutraceutical flavonoids; luteolin (m/z 815.17), quercetagetin (m/z 815.17), kaempferol (m/z 815.17), syringetin (m/z 815.17), limocitrin (m/z 815.17), catechin/epicatechin/pavetannin B2 (m/z 847.19) and helilupolone (m/z 155.1) were detected exclusively in leaf extract. Similarly, two flavonoids; prorepensin (m/z 527.31) and morusin/kuwanon B/cyclomulberrin (m/z 141.06) were only identified in the seed extract, whereas the flavonoid dorsmanin F (m/z 221.1) was detected in both leaf and seed extracts. Flavonoids were not detected in husk extracts. Anthocyanidins and carotenoids, including cyanidin (m/z 816.19), malvidin (m/z 817.23) and rhodopin/OH-lycopene (m/z 555.45), were only identified in the leaf extract. No terpenes were detected in the leaf extract, whereas six and four different terpenes were found in the seed and husk extracts, respectively. Out of six terpenes, five were exclusively detected in seed extract; cinncassiol C (m/z 381.19), ampeloside Bf2 (m/z 985.51), prosapogenin (m/z 663.37), spinasaponin A (m/z 795.44), and medicagenic acid (m/z 413.2). The triterpene saponin, cynarasaponin H (m/z 891.48) was found in both seed and husk extracts. Similarly, three triterpenes; narasin B (m/z 382.25), ginsenoside Rg6 (m/z 384.25) and periandrin I (m/z 855.44) were only identified in the husk extract. A natural phytoalexin, pterostilbene (m/z 221.1) was detected in both leaf and seed extracts, whereas a food-flavoring agent, 2-phenylethanethiol (m/z 139.06) and a phenylpropanoid, coriandrin (m/z 231.07), were identified exclusively in the seed and leaf extracts, respectively. Similarly, metabolites with diverse nutraceutical characteristics, including sarmentine (m/z 204.18), purmorphamine (m/z 261.14), tapentadol (m/z 204.18), zolmitriptan (m/z 270.16), and withaperuvin (m/z 261.14) were only identified in the husk extract.

**Table 3:** Probable metabolites and their possible application/role identified in different plant part of psyllium.

Probable metabolites	Leaves	Seeds	Husk	Possible properties/function/applications
**Alkaloids and flavonoids**				
Lunamarine (punarnavine)	nd	√	nd	Alkaloid (quinolone): anticancer, antiestrogenic, immunomodulatory, and anti-amoebic
Hordatine B	nd	√	nd	Alkaloid: phytoalexin
Pinidine	nd	nd	√	Alkaloid
Luteolin	√	nd	nd	Flavonoid: flavone, nutriceutical, and anticancer
Quercetagetin	√	nd	nd	Flavonoid: flavonol
Kaempferol	√	nd	nd	Flavonoid: flavonol
Syringetin	√	nd	nd	Flavonoid: flavonol
Limocitrin	√	nd	nd	Flavonoid: flavonol
Catechin/epicatechin/pavetannin B2	√	nd	nd	Flavonoid: flavonol
Helilupolone	√	nd	nd	Flavonoid
Dorsmanin F	√	√	nd	Flavonoid: flavanone, antineoplastic activity
Prorepensin	nd	√	nd	Flavonoid
Morusin/kuwanon B/cyclomulberrin	nd	√	nd	Flavonoid: flavone, anti-tumor, and anti-nociceptive activity
**Anthocyanidins and carotenoids**				
Cyanidin	√	nd	nd	Anthocyanidin: antioxidant
Malvidin	√	nd	nd	Anthocyanidin: antioxidant and anticancer (chemopreventive)
Rhodopin/OH-lycopene	√	nd	nd	Carotenoid: antioxidant and singlet oxygen quencher
**Terpenes**				
Cinncassiol C	nd	√	nd	Diterpene: sesquiterpenoid
Narasin B	nd	nd	√	Diterpene glycoside: antibiotic
Ginsenoside Rg6	nd	nd	√	Triterpene glycoside
Ampeloside Bf2	nd	√	nd	Triterpene glycoside: steroidal saponin
Periandrin I	nd	nd	√	Triterpene glycoside; natural sweetener
Prosapogenin	nd	√	nd	Triterpene saponin
Spinasaponin A	nd	√	nd	Triterpene saponin
Cynarasaponin H	nd	√	√	Triterpene saponin
Medicagenic acid	nd	√	nd	Triterpenoid: oleanane triterpenoid
**Others**				
Pterostilbene	√	√	nd	Natural phenol: a phytoalexin
2-Phenylethanethiol	nd	√	nd	Aromatic homomonocyclic compound: a food flavoring agent
Coriandrin	√	nd	nd	Phenylpropanoids and polyketides: exhibits anti-viral function
Sarmentine	nd	nd	√	A natural herbicide
Purmorphamine	nd	nd	√	A therapeutic agent: activates the Hedgehog (Hh) signaling pathway; induce osteoblast differentiation, cardiomyocytes and neuronal differentiation
Tapentadol	nd	nd	√	A therapeutic agent: analgesic
Zolmitriptan	nd	nd	√	A therapeutic agent: used for the acute treatment of migraines
Withaperuvin D/withaperuvin B	nd	nd	√	Withanolide

*nd, not detected; √: present (detected)*.

### Flavonoid Biosynthesis Pathway Analysis

**FIGURE 7:**
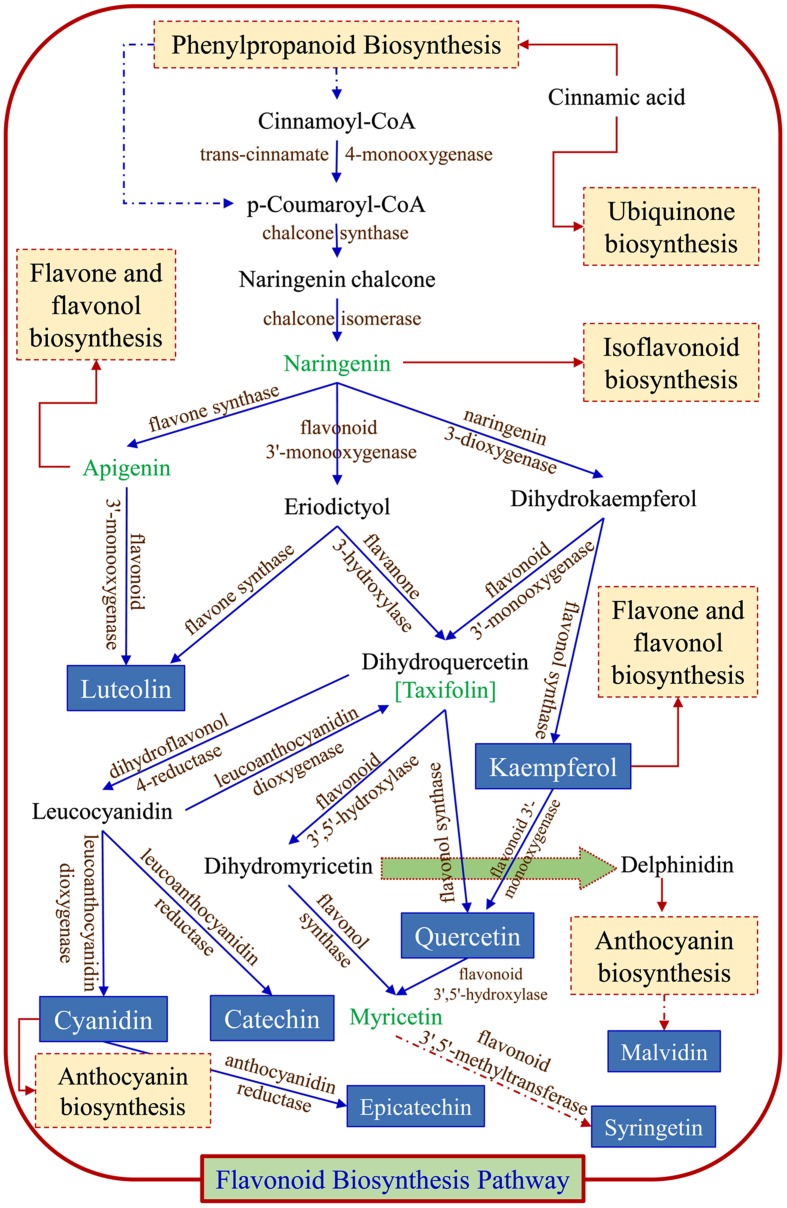
**Probable flavonoid biosynthesis pathway inferred in psyllium.** Blue arrows (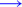) are part of flavonoid biosynthesis pathway; dark red arrows indicate that compound enters in another pathways (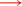), and dotted line/arrows indicate the presence of intermediates.

**FIGURE 8:**
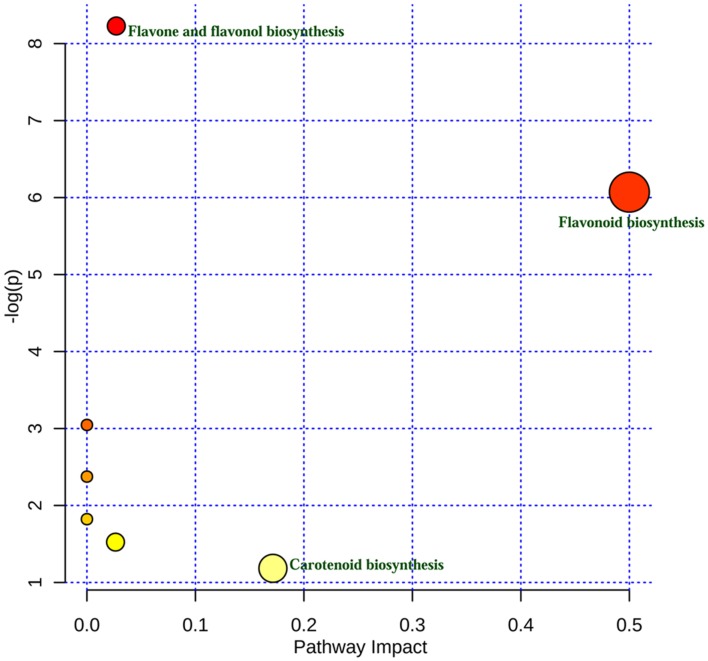
**Interactive graphical output of metabolic pathway analysis**.

Using metabolomic data (Supplementary Table S2), a flavonoid biosynthesis pathway was mapped (**Figure 7**) by *in silico* comparative homology analysis. For this pathway enrichment analysis was performed with the pathway topology using the KEGG metabolic pathways database of *Oryza sativa* japonica and *Arabidopsis thaliana*. The impact of comparative pathway topology analysis showed a maximum interactive degree of centrality with flavonoid biosynthesis (Supplementary Table S4). A graphical output contained three levels of view; metabolome view, pathway view and compound view (**Figure 8**). A probable flavonoid biosynthesis pathway was deduced using the interactive metabolome pathway view that was generated dynamically on the MetaboAnalyst system (**Figure 7**).

## Discussion

*Plantago* species are considered to be a natural reservoir of diverse biologically active secondary metabolites, such as lipids, flavonoids, alkaloids, and terpenoids (Samuelsen, 2000). Psyllium husk (isabgol) is used worldwide as a dietary fiber supplement to relieve constipation, irritable bowel syndrome and diarrhea. Furthermore, it has also been recognized as a cholesterol-lowering agent for use in hypercholesterolemia (Anderson et al., 1990). In recent years, natural antioxidants from different plant sources have gained increasing attention (Mishra et al., 2015) and *P. ovata* plant tissues are rich in bioactive compounds, including different types of metabolites, such as polyphenols and flavonoids (Talukder et al., 2015), which are well-known natural antioxidants.

Essential FAs, including alpha-linolenic acid (a ω-3 fatty acid) and linoleic acid (a ω-6 fatty acid) cannot be synthesized *de novo* in sufficient quantity in humans and is therefore outsourced by the human body from food (Food and Agriculture Organization, 2010). A bi-plot data matrix of the total lipids and fatty acids successfully explained 100% of the variance (PC1 – 55.27% and PC2 – 44.73%) and revealed correlations between FAs and plant parts (**Figure 2**). Overall, the PCA exhibited a statistical distinction among the total lipids and fatty acids composition of different plant parts. In the present study, different psyllium plant parts, such as leaves, seeds and the husk were found to be a rich source of essential ω-3 and ω-6 fatty acids that are involved in human physiology (**Table 1**). About 14.57, 17.39, and 0.76 mg g^-1^ alpha-linolenic acid (ALA, ω-3 FA) were detected in leaves, seeds and the husk respectively (Supplementary Table S3). Seed oils are considered rich source of ALA, which is one of the essential fatty acid and cannot be produced within the human body. Additionally, psyllium was found to be a rich source of PUFAs, which are imperative nutritional indicators that demonstrate the nutraceutical importance of the plant (Gill and Valivety, 1997a). Furthermore, PUFAs have a number of non-edible biotechnological applications, including drying oils and biotransformations (Gill and Valivety, 1997b). In addition to fatty acids, a higher content of the essential amino acid valine was found in psyllium leaves followed by sulfur-rich amino acids (**Figure 4**), compared to the FAO recommended reference pattern (Food and Agriculture Organization, 2013). The essential amino acid leucine and the sulfur-rich amino acids cysteine and methionine were not detected in the husk. This implies that psyllium leaves are a rich source of essential amino acid (valine) and fatty acids (ω-3 fatty acid, alpha-linolenic acid and ω-6 fatty acid, linoleic acid) and can therefore act as a dietary supplement. Ideal daily fat intake is 55–83 g, which is 20–30% (500–700 calories) of daily energy requirement, i.e., 2500 calories (Food and Board, 1989). Results demonstrated that 100 g psyllium leaves contain 2.62 g fat, in result about 24 calories energy. There are 0.24 and 0.15 g fat in 100 g of mixed salad greens and Lettuce green leaves salad, which give 2.2 and 1.4 calories in the term of fat, respectively. In total (including fat, carbohydrate and proteins), there is 17 calories in 100 g of mixed salad greens, whereas 15 calories in 100 g of Lettuce green leaves salad (USDA, 2014). The recommended daily intake of essential amino acids, histidine, isoleucine, leucine, lysine, threonine, and valine are 10, 20, 39, 30, 15, 26 mg/kg-weight/day, respectively for adults (Food and Board, 1989). Psyllium leaves contain 0.28, 1.17, 0.93, 0.27, 0.95, and 2.79 g histidine, isoleucine, leucine, lysine, threonine, and valine per 100 g of biomass, respectively. About 100 g leaves provide 56, 48, and 18% daily requirement of histidine, leucine, and lysine to an adult of an average weight 50 kg. Furthermore, 100 g leaves fulfill more than recommended daily requirement of isoleucine (117%), threonine (127%), and valine (215%). Taken together, daily fat intake and essential amino acids, psyllium leaves can be used as a green salad together with daily food as a dietary supplement.

Phytochemicals (secondary metabolites), including flavonoids, phenolic acids and polyphenols, are potent antioxidants are ubiquitous in plants and are an essential part of the human diet (Embuscado, 2015; Mishra et al., 2015). Psyllium seeds contained a higher total phenolic content than leaves and the husk (**Figure 5**), and therefore show a higher total antioxidant and DPPH scavenging activity, followed by leaves and the husk (**Figures 6A,B**). The maximum total flavonoid content was detected in leaves followed by seeds and the husk (**Figure 5**); leaves also possess high superoxide scavenging activity, whereas the husk showed the maximum reducing activity (**Figures 6C,D**). Thus, a positive correlation was observed between the phenolic and flavonoid contents, and the antioxidant and scavenging activities of psyllium plant parts. Secondary metabolites are considered to be efficient antioxidants and important radical scavengers, and also possess biological activities (Shahidi and Ambigaipalan, 2015). Antioxidant activities inhibit oxidation processes of functional food ingredients and thus, play a key role in promoting health (Shahidi and Ambigaipalan, 2015). Scavenging and antioxidant activities depend on the content of polyphenolics and flavonoids (Shahidi and Ambigaipalan, 2015). Similar to the finding in this study, a direct correlation was observed between scavenging and antioxidant activities, and the content of polyphenolics and flavonoids in Cumin, Salicornia, and Plantago (Mishra et al., 2015; Pandey et al., 2015; Talukder et al., 2015). The total antioxidant and DPPH scavenging activity increased concomitantly with the polyphenolic and flavonoid content during different stages of *in vitro* callus culture of *P. ovata* (Talukder et al., 2015). Similarly, a correlation factor in terms of reciprocal values was established between the total phenolic and flavonoid content, and the antioxidant activity of shoot extracts of some selected *Plantago* species (Beara et al., 2009).

Previous studies have revealed that *Plantago* species are an excellent source of secondary metabolites with widespread applications in the nutraceutical industry (Samuelsen, 2000; Beara et al., 2009), and in the present study, about 13 different metabolites were identified in leaf and seed extract of psyllium, whereas 10 metabolites were found in the husk (**Table 3**). This study is the first report on the untargeted metabolomics of different psyllium (*P. ovata*) plant parts (leaves, seeds, and husk). Most flavonoids were detected in psyllium leaf extract, whereas terpenes were abundant in the seed extract. Flavonoids perform distinctive functions in plant metabolic pathways, including pigmentation, nutrition and defense (Horborne and Williams, 2000). Metabolites, apigenin, luteolin, rutin, quercetin, scutellarein, flavonoids, and triterpene acids were reported from leaves of *Plantago* species, especially from *P. major*, for a range of biological activities, including wound healing, anti-inflammatory, analgesic, antioxidant, weak antibiotic, immune-modulating and antiulcerogenic activity (Kawashty et al., 1994; Samuelsen, 2000; Beara et al., 2009; Gonçalves and Romano, 2016). Furthermore, plant leaves were also used to cure asthma, bronchitis, pulmonary diseases, and diabetes (Samuelsen, 2000; Gonçalves and Romano, 2016).

Most metabolites, including lunamarine, luteolin, quercetagetin, kaempferol, syringetin, catechin, epicatechin, helilupolone, cyanidin, malvidin and quercetin are phenolics, flavonoids or alkaloids and contain potent antioxidant activities (Grotewold, 2006; Gould and Lister, 2006; Mishra et al., 2015). These metabolites are known to be natural antioxidants and nutrient supplements, and provide functional value for the plant by modulating cell-signaling pathways (Williams et al., 2004). Plant flavonoids are divided into six major groups; chalcones, flavones, flavonols, flavandiols, anthocyanins, and condensed tannins (or proanthocyanidins); whereas a seventh group, aurones, is also present, but is not ubiquitous (Winkel-Shirley, 2001). Moreover, isoflavonoids are commonly synthesized by legumes. The alkaloid, lunamarine or punarnavine was detected exclusively in psyllium seed extract and displays a number of pharmaceutical applications, including anti-cancer, anti-estrogenic, immunomodulatory and anti-amoebic activities (Manu and Kuttan, 2009; Sreeja and Sreeja, 2009). Another alkaloid, hordatine B, which is ubiquitous in barley (Smith and Best, 1978), was also detected only in the seed extract, and is a well-known phytoalexin that confers antifungal activity to germinating seeds (Stoessl and Unwin, 1970).The flavonoids morusin and prorepensin were detected exclusively in seed extracts and are reported to be a potent antitumor agent and antioxidant, respectively (Tseng et al., 2010). The flavonoid, dorsmanin F was found in leaf and seed extracts, and is known for its anti-neoplastic activity (Kuete et al., 2015). The flavonoids luteolin and quercetin, which were only detected in psyllium leaf extracts, show potential for cancer prevention and therapy (Lin et al., 2008; Zhang et al., 2014). A potent antioxidant, kaempferol, known for a wide range of pharmacological activities, is used for numerous preclinical studies, due to its antioxidant, anti-inflammatory, anti-microbial, anti-cancer, cardioprotective, neuroprotective, antidiabetic, anti-osteoporotic, estrogenic/antiestrogenic, anxiolytic, analgesic and antiallergic activities (Calderon-Montano et al., 2011). The flavonoid syringetin is reported to stimulate osteoblast differentiation (Hsu et al., 2009), whereas other flavonoids, including limocitrin, catechin/epicatechin, pavetannin, helilupolone, kuwanon B, and cyclomulberrin contain antioxidant activities. Anthocyanidins, including cyanidin, malvidin, and rhodopin/OH-lycopene were detected exclusively in psyllium leaf extracts and are natural antioxidants. Antioxidant metabolites (flavonoids and anthocyanidins) were predominant in psyllium leaf extracts and therefore, plant leaves can be further explored in terms of their antioxidants. Psyllium seed and husk extracts contained terpenes, including saponins (**Table 3**), and previous studies demonstrated that saponins might possess anticancer activity (Gurfinkel and Rao, 2003; Zhu et al., 2005). Psyllium seed extract was observed to be a potent source of saponins (terpenes) and saponins are thought to contribute natural plant defenses against pathogens and to act as scavengers of ROS; additionally, they have wide applications in the food, cosmetic and pharmaceutical industries (Güçlü-Üstündaǧ and Mazza, 2007).

In plants, the flavonoid biosynthesis pathway has gained importance throughout the world and it is one of the most intensively studied pathways (Grotewold, 2006). Flavonoid biosynthesis was developmentally or environmentally controlled by transcriptional regulatory networks; MYB–bHLH–WDR complexes, which are well-conserved in higher plants (Xu et al., 2015). In this study, the existence of a potential flavonoid biosynthesis pathway was inferred (**Figure 7**) using metabolomic data and a pathway topology module by *in silico* comparative homology analysis. The pathway includes a probable route for the biosynthesis of metabolites that were detected in different plant parts (**Table 3**). Furthermore, the nexus of different pathways such as the flavone and flavonol, isoflavonoid, anthocyanin and ubiquinone biosynthesis pathways was also observed. The flavonoid biosynthesis was started with general phenylpropanoid metabolism and leading to the major subgroup pathways; flavones and flavonol, isoflavonoid, and anthocyanin biosynthesis pathways. Metabolites, apigenin and kempferol lead to flavones and flavonol pathway, whereas cyanidin and delphinidin enter into anthocyanin biosynthesis pathway. Metabolite naringenin intermediates between flavonoid and isoflavonoid biosynthesis pathway. The major metabolites end products are luteolin, catechin, epicatechin, syringetin, kempferol, cyanidin, and quercetin, which were also detected by LC MS analysis. The proposed illustration provides a paradigm to understand the transcriptional regulation of flavonoid biosynthesis and to engineer metabolic pathways accordingly to the demands of the nutraceutical industry.

## Conclusion

This study reveals that psyllium (*P. ovata* Forsk) contains nutritional antioxidants, flavonoids, PUFAs, including essential fatty acids (ω-3 and ω-6 fatty acids), sulfur-rich and essential amino acids, and metabolites with bioactivities, which make it a promising candidate for use in the nutraceutical industry. Additionally, psyllium leaves can be used as a green salad together with daily food as a dietary supplement. As a future perspective, the flavonoid biosynthesis pathway illustrated here provides useful insight and opens a new avenue to select regulatory gene(s) for metabolic engineering.

## Author Contributions

Conceived and designed the experiments: AM and BJ. Performed the experiments: MP. Analyzed the data: MP and AM. Wrote the paper: MP and AM.

## Conflict of Interest Statement

The authors declare that the research was conducted in the absence of any commercial or financial relationships that could be construed as a potential conflict of interest. The reviewer AG and handling Editor declared their shared affiliation, and the handling Editor states that the process nevertheless met the standards of a fair and objective review.
