# Erratum: Non-targeted Metabolite Profiling and Scavenging Activity Unveil the Nutraceutical Potential of Psyllium (*Plantago ovata* Forsk)

**DOI:** 10.3389/fpls.2016.00614

**Published:** 2016-05-06

**Authors:** Frontiers Production Office

**Affiliations:** Frontiers Production Office, FrontiersSwitzerland

**Keywords:** antioxidant, bioactivity, isabgol, medicinal plant, metabolomics

Reason for Erratum:

Due to a typesetting error, the abbreviation “CPS” was introduced several times throughout the text, and should not be part of the manuscript.

The publisher apologizes for this mistake and the original article has been updated.

This error does not change the scientific conclusions of the article in any way.

