# Supplementary material for: Non-targeted Metabolite Profiling and Scavenging Activity Unveil the Nutraceutical Potential of Psyllium (Plantago ovata Forsk)
Source: Front Plant Sci. 2016 Apr 5;7:431. doi: 10.3389/fpls.2016.00431 (PMC4821064; doi:10.3389/fpls.2016.00431)
Supplement: Supplementary file 1 [file Table_1.PDF]

Supplementary Material:

Non-targeted metabolite profiling and scavenging activity unveil nutraceutical potential of psyllium *(Plantago ovata Forsk)*

Manish Kumar Patel, Avinash Mishra* and Bhavanath Jha*
*Corresponding authors: avinash@csmcri.org (AM) and bjha@csmcri.org (BJ)

Table S1: Estimated LOD and LOQ of fatty acids for GCMS model QP2010

Fatty acids	Common Name	LOD (mg/ml)	LOQ (mg/ml)
C12:0	Lauric acid	0.02500	0.07500
C14:0	Myristic acid	0.00624	0.01872
C14:1	Myristoleic acid	0.01250	0.03750
C15:0	Pentadecanoic acid	0.00624	0.01872
C15:1 (n-5)	Cis-10-Pentadecenoic acid	0.00624	0. 01872
C16:0	Palmitic acid	0.00468	0.01404
C16:1 (n-7)	Palmitoleic acid	0.00624	0.01872
C17:0	Heptadecanoic acid	0.00312	0.00936
C17:1 (n-7)	Cis-10-Heptadecenoic acid	0.00312	0.00936
C18:0	Stearic acid	0.00312	0.00936
C18:1 (n-9)	Oleic acid	0.00624	0.01872
C18:2 (n-6)	Linoleic acid	0.00312	0.00936
C18:3 (n-3)	α-Linolenic acid	0.00312	0.00936
C18:3 (n-6)	γ-Linolenic acid	0.00312	0.00936
C20:0	Arachidic acid	0.02500	0.07500
C20:1 (n-9)	Cis-11-Eicosenoic acid	0.01250	0.03750
C20:2	Cis-11,14-Eicosadienoic acid	0.00312	0.00936
C20:3 (n-3)	Cis-11,14,17-Eicosatrienoic acid	0.02500	0.07500
C21:0	Henicosanoic acid	0.00312	0.00936
C22:0	Behenic acid	0.00624	0.01872
C22:1 (n-9)	Erucic acid	0.00156	0.00468
C23:0	Tricosanoic acid	0.00624	0.01872
C24:0	Lignoceric acid	0.00624	0.01872

Table S2: Metabolite used in pathway analysis and outcome to be used for pathway mapping

Query	Match	HMDB	PubChem	KEGG
Lunamarine	Linamarin	HMDB33699	11128	C01594
Punarnavine	NA	NA	NA	NA
Cryptodorine	Cryptodorine	HMDB33359	11438278	
Piperolactam C	Piperolactam C	HMDB38584	10881419	
Hordatine B	Hordatine B	HMDB30459	NA	C08308
Pinidine	NA	NA	NA	NA
Luteolin	Luteolin	HMDB05800	5280445	C01514
Quercetagitrin	NA	NA	NA	NA
Kaempferol	Kaempferol	HMDB05801	5280863	C05903
Syringetin	Syringin	HMDB08053	52922667	C00157
Limocitrin	Limocitrin	HMDB29516	5489485	
Catechin	Catechin	HMDB02780	9064	C06562
Epicatechin	Epicatechin	HMDB01871	72276	C09727
Pavetannin B2	Pavetannin B2	HMDB39572	13990886	
Helilupolone	NA	NA	NA	NA
Dorsmanin F	NA	NA	NA	NA
Prorepensin	NA	NA	NA	NA
Morusin	Morusin	HMDB36631	5281671	C10106
Kuwanon B	Kuwanon B	HMDB29506	44258295	
Cyclomulberrin	Cyclomulberrin	HMDB30688	11742872	
Cyanidin	Cyanidin	HMDB02708	68247	C05905
Malvidin	Malvidin	HMDB03201	69512	C08716
Rhodopin	NA	NA	NA	NA
Lycopene	Lycopene	HMDB03000	446925	C05432

Table S3: Fatty acid content (in mg g^{-1}) of different psyllium plant parts

Fatty acids	Name of fatty acids	Leaf	Seed	Husk
C12:0	Lauric acid	nd	nd	0.28
C14:0	Myristic acid	nd	0.40	0.07
C14:1	Myristoleic acid	0.21	nd	nd
C15:0	Pentadecanoic acid	0.03	0.21	0.03
C15:1 (n-5)	Cis-10-Pentadecenoic acid	nd	0.08	nd
C16:0	Palmitic acid	4.15	9.09	3.48
C16:1 (n-7)	Palmitoleic acid	0.03	0.27	0.03
C17:0	Heptadecanoic acid	0.04	0.35	0.03
C17:1 (n-7)	Cis-10-Heptadecanoic acid	nd	0.22	0.02
C18:0	Stearic acid	1.31	9.34	1.37
C18:1 (n-9)	Oleic acid	0.50	nd	5.34
C18:2 (n-6)	Linoleic acid	4.61	100.10	12.72
C18:3 (n-3)	alpha-Linolenic acid [ALA]	14.57	17.39	0.76
C18:3 (n-6)	gamma-Linolenic acid	nd	0.60	0.12
C20:0	Arachidic acid	0.10	0.98	0.16
C20:1 (n-9)	Cis-11-Eicosenoic acid	nd	12.01	0.58
C20:2	Cis-11,14-Eicosadienoic acid	nd	nd	1.32
C20:3 (n-3)	Cis-11,14,17-Eicosadienoic acid	0.28	nd	nd
C21:0	Heneicosanoic acid	nd	0.71	0.11
C22:0	Behenic acid	0.12	0.40	0.11
C22:1 (n-9)	Erucic acid	nd	nd	0.12
C23:0	Tricosanoic acid	nd	0.13	nd
C24:0	Lignoceric acid	0.22	0.24	nd

Amount in mg g^{-1} of biomass

Table S4: The impact of comparative pathway topology analysis

Pathways	Total	Expected	Hits	Raw p	- log(p)	Holm adjust	FDR	Impact
Flavonoid biosynthesis	37	0.35796	4	0.000267	8.229	0.022145	0.022145	0.50
Flavone and flavonol biosynthesis	8	0.077397	2	0.00231	6.0705	0.18942	0.095862	0.03
Linoleic acid metabolism	5	0.048373	1	0.047528	3.0464	1	1	0.00
Arachidonic acid metabolism	10	0.096746	1	0.092994	2.3752	1	1	0.00
alpha-Linolenic acid metabolism	18	0.17414	1	0.16165	1.8223	1	1	0.00
Glycerophospholipid metabolism	25	0.24186	1	0.21781	1.5241	1	1	0.03
Carotenoid biosynthesis	37	0.35796	1	0.30619	1.1836	1	1	0.17
